# Outcomes of dengue infection in adults with underlying haematological diseases in Brazil during 2024 and 2025

**DOI:** 10.1111/bjh.70527

**Published:** 2026-05-19

**Authors:** K. Tozatto‐Maio, V. Weihermann, C. W. Erthal, M. W. Araújo, C. R. Gomes, K. Caciola, L. Otuyama, J. Gasparini, H. R. Higashino, S. F. Costa, A. N. Duarte‐Neto, F. S. Seguro, P. R. Villaça, E. D. R. P. Velloso, S. F. M. Gualandro, Y. Nukui, V. Rocha

**Affiliations:** ^1^ Laboratório de Investigação Médica 31 (LIM31) Hematology and Cell Therapy Service, Hospital das Clínicas da Faculdade de Medicina da Universidade de São Paulo São Paulo Brazil; ^2^ Einstein Hospital Israelita São Paulo Brazil; ^3^ NHS University Hospital University of Oxford Oxford UK

**Keywords:** dengue fever, haematological diseases, neglected tropical disease

To the Editor,

Dengue fever (DF) is a major re‐emerging mosquito‐borne viral disease caused by four dengue virus (DENV) serotypes (DENV‐1 to DENV‐4) and transmitted primarily by *Aedes aegypti*. Its global burden has risen substantially due to urbanization, globalization and climate change.^1^, ^2^ According to the World Health Organization (WHO), reported dengue cases increased from approximately 500 000 in 2000 to over 14 million cases and more than 10 000 deaths in 2024.^3^ Brazil experienced an unprecedented epidemic in 2024–2025.^4^ WHO data indicated more than 6.3 million suspected cases (3.0 million confirmed) in 2024, the highest number reported worldwide, with simultaneous circulation of all four serotypes.^3^

The clinical spectrum of DF ranges from asymptomatic infection to severe disease characterized by plasma leakage, bleeding and organ dysfunction. Increased vascular permeability is the hallmark of severe dengue and may result in hypovolaemic shock, haemoconcentration and serosal effusions.^5^ This capillary leak syndrome arises from interactions among immune activation, endothelial dysfunction and viral factors.

Patients with haematological diseases may be particularly vulnerable to severe or atypical dengue presentations. Pre‐existing cytopenias, haemolysis or bone marrow dysfunction may overlap with dengue‐induced abnormalities, complicating diagnosis and management. Case reports have described DF in individuals with sickle cell disease (SCD) (homozygous and compound heterozygous), autoimmune haemolytic anaemia, immune thrombocytopenia, paroxysmal nocturnal haemoglobinuria and myelodysplastic syndromes (MDS), suggesting synergistic effects that increase the risk of severe complications.^6^, ^7^, ^8^

Despite these concerns, data on dengue outcomes in patients with haematological disorders remain scarce. As dengue is classified as a neglected tropical disease, disproportionately affecting low‐income populations, the literature is limited to isolated case reports or small retrospective series, limiting evidence‐based guidance. Reporting outcomes in this population is therefore essential to improve clinical recognition, enable early identification of complications and support tailored supportive care. This retrospective, observational study describes the characteristics and outcomes of DF in 36 adult patients followed at the Instituto Central do Hospital das Clínicas, Faculdade de Medicina da Universidade de São Paulo (ICHC‐FMUSP). The Hematology Day Hospital provides outpatient care for patients with red blood cell and haemostasis disorders, myeloproliferative syndromes, aplastic anaemia and MDS. All patients who were admitted to the Day Hospital with symptoms and had a confirmed case of DF during the study period were included in the analyses.

Patients presenting with symptoms suggestive of DF underwent diagnostic testing with non‐structural protein 1 (NS1) antigen and/or immunoglobulin M(IgM)/immunoglobulin G (IgG) serology (Bioline™ DENGUE DUO kit, Abbott®). Test selection was based on symptom duration: NS1 was preferentially performed within the first 5 days of symptoms, whereas IgM/IgG was prioritized from day 6 onwards; in some cases, both were obtained. Laboratory evaluation at presentation included complete blood count, liver enzymes and renal function tests.

Cases were classified and managed according to Brazilian Ministry of Health dengue management guidelines, which stratify clinical severity from group A to group D based on comorbidities, bleeding manifestations and dehydration.^9^ Hospital admission was indicated in the presence of clinical or laboratory deterioration, including haemodynamic instability, severe dehydration, worsening cytopenia or progressive elevation of liver enzymes. In all cases, hospitalization was decided based on the criteria from the Brazilian Ministry of Health, namely classification in group C or D and acute decompensation of the underlying disease, as indicated in the Table S1. Decisions regarding threshold levels for platelet transfusions followed the American Association of Blood Banks (AABB) guidelines.^10^ Patients received red blood cell transfusions if they presented symptomatic anaemia or, in the case of SCD with severe complications, to lower haemoglobin S levels.

Data were collected using REDCap and analysed in R v3.3.4. The study was approved by the local ethics committee (CAAE 81434524.4.0000.0068) and informed consent was obtained. Artificial intelligence has not been used to develop this work.

Between March 2024 and June 2025, 48 patients presented with suspected DF; 36 had confirmed infection (positive NS1 and/or IgM) and were included in the analysis. Nineteen patients were female, and the median age was 38 years (range, 19–60).

SCD was the most common underlying diagnosis (*n* = 13; 10 SS genotype, 2 Sβ^0^ genotype and 1 SC genotype). Other haematological conditions included MDS (*n* = 1), aplastic anaemia (*n* = 6), myeloproliferative neoplasms (*n* = 4; 2 polycythaemia vera and 2 essential thrombocythaemia), immune thrombocytopenia (*n* = 3), haemophilia (*n* = 2), anti‐phospholipid syndrome (*n* = 2), xerocytosis (*n* = 1), paroxysmal nocturnal haemoglobinuria (*n* = 1), autoimmune haemolytic anaemia with autoimmune neutropenia (*n* = 1) and pure red cell aplasia (*n* = 1). One case of transfusion‐transmitted dengue occurred in an autologous haematopoietic stem cell transplant recipient, confirmed by positive polymerase chain reaction in the patient and in the red blood cell product. Ten patients had underlying liver disease (predominantly secondary haemochromatosis; one had a prior liver transplant), and three had chronic kidney disease.

The most frequent presenting symptoms were fever (*n* = 32), myalgia (*n* = 30), headache (*n* = 23), arthralgia (*n* = 19), vomiting (*n* = 11) and retro‐orbital pain (*n* = 11). Median time between onset of symptoms and admission to our centre was 4 days (range, 1–8). During initial evaluation, 29 patients were classified as group B, 6 as group C and 1 as group D. Bleeding manifestations occurred in seven patients, abdominal pain in six and rash in three. No cavitary effusions were present at admission. NS1 antigen testing was performed in 34 patients and was positive in 32; IgM serology was performed in 27 and was positive in 13. Twenty‐five patients had both NS1 and IgM serology performed.

During disease progression, liver failure occurred in three patients, bleeding episodes in eight, hypovolaemic shock in two and cavitary effusion (ascites) in one patient who had a previous liver transplant due to Budd–Chiari syndrome. Thirteen patients required red blood cell transfusions, and five required platelet transfusions.

Eleven patients (30%) required hospitalization. Six were male, six had SCD and five had underlying liver disease. Two SCD (SS genotype) patients required intensive care. One patient was hospitalized at day 5 of symptoms due to worsening of generalized pain. At admission, she presented leucocytosis with a left shift in the blood film, haemoglobin was 1 g/dL lower than baseline and lactate dehydrogenase was elevated. The next day, she developed multiorgan failure, had a sudden abdominal pain followed by cardiac arrest and died. Her autopsy revealed bone marrow necrosis, pulmonary thromboembolism and bone marrow emboli in lung vessels (Figure S1). The second patient developed ischaemic hepatitis and secondary bacterial infection, required mechanical ventilation, vasopressors and renal replacement therapy, and was discharged after 31 days of hospitalization.

Laboratory data stratified by diseases (SCD, aplastic anaemia/MDS and myeloproliferative syndromes) collected at day 0 and day 5 from first admission are presented in Table 1. Of note, no haematological patient presented haematocrit values above 44% for women and 50%, a known severity marker in DF. Only three patients had laboratory data collected on day 10, and none of them had abnormal values compared with baseline.

**TABLE 1:** Laboratory exams collected at D0 and D5 from admission—median (min–max).

Haematological diagnosis	D0			D5		
	SCD	AA + MDS	MPS	SCD	AA + MDS	MPS
	*N* = 10	*N* = 7	*N* = 3	*N* = 7	*N* = 2	*N* = 1
Haemoglobin (g/dL)	8.50 (6.10–10.10)	7.70 (5.30–14.90)	13.10 (12.80–15.50)	6.95 (5.60–9.00)	7.80 (7.80–7.80)	11.90 (11.90–11.90)
Haematocrit (%)	24 (15–27)	20 (14–37)	38 (32–39)	20.3 (14.6–25.7)	22.2 (22.2–22.2)	35.0 (35.0–35.0)
White blood cell count (WBC) (10^9^/L)	7.1 (1.2–17.7)	1.1 (0.1–2.6)	1.5 (0.8–1.9)	8.1 (3.1–12.1)	0.3 (0.3–0.3)	1.4 (1.4–1.4)
Neutrophils (10^9^/L)	4.1 (0.7–13.8)	0.6 (0.0–1.4)	0.7 (0.4–1.1)	2.45 (1.07–5.37)	—	0.72 (0.72–0.72)
Lymphocytes (10^9^/L)	1.70 (0.40–2.40)	0.55 (0.00–1.00)	0.50 (0.30–0.63)	4.22 (1.88–6.30)	—	0.44 (0.44–0.44)
Platelets (10^9^/L)	247 (45–492)	23 (2–53)	213 (84–226)	60 (40–217)	9 (9–9)	105 (105–105)
Reticulocytes (10^9^/L)	107 (47–185)	—	26 (26–26)	22 189 (47–44 330)	—	—
Aspartate aminotransferase (AST) (mg/dL)	179 (41–1267)	130 (32–362)	38 (30–115)	98 (50–146)	—	48 (48–48)
Alanine aminotransferase (ALT) (mg/dL)	74 (8–832)	127 (15–274)	34 (33–79)	34 (15–53)	—	40 (40–40)
Total bilirubin (mg/dL)	3.79 (0.78–11.66)	0.75 (0.48–1.34)	0.33 (0.31–0.35)	4.11 (0.71–6.84)	—	0.30 (0.30–0.30)
Direct bilirubin (mg/dL)	0.65 (0.30–2.60)	0.30 (0.10–0.80)	0.20 (0.10–0.20)	0.40 (0.40–1.60)	—	0.20 (0.20–0.20)
Indirect bilirubin (mg/dL)	1.40 (0.50–11.10)	0.40 (0.30–1.00)	0.20 (0.20–0.20)	3.70 (0.30–5.20)	—	0.10 (0.10–0.10)
Alkaline phosphatase (mg/dL)	79 (49–1249)	101 (73–482)	58 (51–92)	59 (49–69)	—	54 (54–54)
Gama‐glutamil transferase (GGT) (mg/dL)	62 (17–474)	76 (15–854)	116 (62–139)	43 (39–46)	—	51 (51–51)
C‐reactive protein (mg/L)	22 (4–306)	42 (10–119)	3 (3–3)	94 (94–94)	49 (49–49)	12 (12–12)
Lactate dehydrogenase (LDH) (U/L)	878 (466–1666)	603 (305–901)	—	647 (591–674)	—	—
Creatinine (mg/dL)	0.69 (0.43–1.31)	0.87 (0.45–1.17)	0.85 (0.76–1.66)	0.85 (0.37–1.14)	—	3.36 (3.36–3.36)
Urea (mg/dL)	27 (14–71)	26 (16–32)	19 (10–68)	18 (14–21)	—	151 (151–151)

Abbreviations: AA, aplastic anaemia; MDS, myelodysplastic syndrome; MPS, myeloproliferative syndrome; SCD, sickle cell disease.

This study represents one of the few adult series describing DF in patients with underlying haematological diseases and highlights the diagnostic and clinical challenges in this population. Dengue warning signs rely partly on haematological parameters; however, cytopenias and other laboratory abnormalities are often chronic baseline findings in haematological disorders, potentially masking early signs of clinical deterioration and contributing to delayed recognition of DF as a severe disease. An approach based on individual trends might benefit this population; however, studies with larger cohorts are warranted to define.

Consistent with prior literature, patients with SCD experienced the most severe complications. Two required intensive care, and one patient died. Multiple immunopathological mechanisms likely contribute to this vulnerability. SCD is characterized by chronic inflammation, monocyte activation, endothelial dysfunction and microvascular occlusion,^11^ processes that may be further amplified by dengue‐associated endothelial injury and capillary leak.^11^, ^12^ The occurrence of bone marrow necrosis in one patient represents a particularly severe and rare complication; to our knowledge, this is the first reported case of bone marrow necrosis associated with DF. Previous studies, largely in paediatric populations, have similarly demonstrated poor outcomes in SCD patients, including a case fatality rate of 12.5% among SC or SS individuals compared with 0.41% in the general population,^6^ and severe dengue in 47.1% of 102 patients in another series.^7^ In contrast, 26% of patients in the present cohort required hospitalization, markedly higher than the approximate 3.6% hospitalization rate reported among nearly 14.9 million dengue cases reported in Brazil from 2014 to 2024.^13^

Data on dengue infection in other haematological diseases remain limited. A small case series of five patients reported no mortality and no worsening of the underlying haematological disease,^14^ whereas a recent abstract describing 52 individuals with haematological malignancies or post–haematopoietic stem cell transplantation reported hospital admission in 75%, intensive care in 15% and three deaths.^8^ Our findings similarly suggest increased risk among patients with marrow failure syndromes, chronic cytopenias or underlying liver disease, emphasizing the importance of close monitoring, cautious fluid management and timely transfusion support.

Although there is only one documented case of transfusion‐related DF, most patients in this cohort might need transfusion, either occasionally or chronically. To date, screening for dengue virus is not routinely performed in blood donors.^15^ Furthermore, currently available dengue vaccines are not recommended for immunocompromised individuals, such as transplant recipients,^16^ as they are based on live attenuated viral platforms.^17^ Considering the potentially severe clinical course of DF in this population, further studies assessing the risk of transfusion‐related DF might be necessary to discuss the implementation of pre‐transfusion dengue virus screening.

This study has limitations, including its retrospective design and heterogeneity of haematological disorders. The small sample size limited the robustness of statistical analyses, thereby restricting the generalizability of the results. Another key limitation of this study is the lack of an institutional control group comparable to the general population, which would help minimize biases associated with historical comparisons. However, as our hospital exclusively manages highly complex cases, such a control group is not feasible. Larger, multicentre studies are needed to better define risk factors and inform evidence‐based management strategies. As dengue continues to expand globally,^1^, ^2^, ^3^ identifying high‐risk populations and implementing tailored clinical strategies are increasingly important. Early recognition of warning signs, close monitoring and individualized supportive care may be critical to improving outcomes in this vulnerable population.

## AUTHOR CONTRIBUTIONS

**V. Weihermann:** Writing – original draft; writing – review and editing; data curation. **C. W. Erthal:** Data curation; writing – review and editing. **J. Gasparini:** Formal analysis. **A. N. Duarte‐Neto:** Data curation; writing – review and editing. **S. F. Costa:** Data curation; writing – review and editing. **F. S. Seguro:** Data curation; writing – review and editing. **C. R. Gomes:** Data curation; writing – review and editing. **K. Caciola:** Data curation; writing – review and editing. **P. R. Villaça:** Data curation; writing – review and editing. **M. W. Araújo:** Data curation; writing – review and editing. **K. Tozatto‐Maio:** Conceptualization; writing – original draft; methodology; writing – review and editing; data curation. **H. R. Higashino:** Data curation; writing – review and editing. **L. Otuyama:** Formal analysis. **E. D. R. P. Velloso:** Data curation; writing – review and editing. **V. Rocha:** Conceptualization; writing – original draft; writing – review and editing; supervision. **S. F. M. Gualandro:** Data curation; supervision; writing – review and editing. **Y. Nukui:** Data curation; supervision; writing – review and editing.

## FUNDING INFORMATION

This work has not been funded by any sources.

## CONFLICT OF INTEREST STATEMENT

The authors have no conflicts of interest to disclose.

## ETHICS STATEMENT

The study was approved by the local ethics committee (CAAE 81434524.4.0000.0068).

## PATIENT CONSENT STATEMENT

The authors confirm that written informed consent has been obtained from the involved patients.

## Supporting information


Figure S1.

Table S1.

Appendix S1.

## Data Availability

Data are available upon request.
