# Supplementary material for: Outcomes of dengue infection in adults with underlying haematological diseases in Brazil during 2024 and 2025
Source: Br J Haematol. 2026 May 19;209(1):324–8. doi: 10.1111/bjh.70527 (PMC13340477; doi:10.1111/bjh.70527)
Supplement: Supplementary file 1 — Figure S1. [file BJH-209-324-s002.docx]

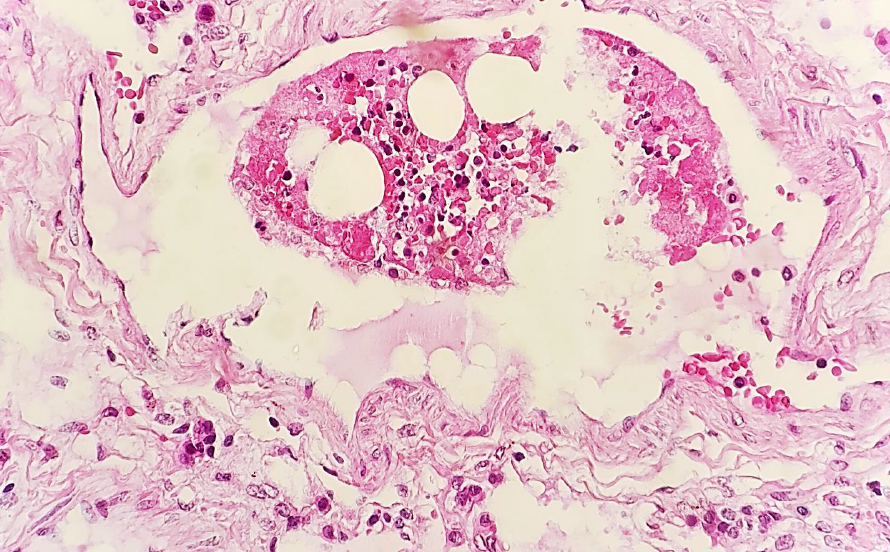


Supplementary figure 1: Hematoxylin and eosin-stained slide: large size pulmonary vessel with a thrombus containing bone marrow elements (hematopoietic cells)
