# Supplementary material for: Outcomes of dengue infection in adults with underlying haematological diseases in Brazil during 2024 and 2025
Source: Br J Haematol. 2026 May 19;209(1):324–8. doi: 10.1111/bjh.70527 (PMC13340477; doi:10.1111/bjh.70527)
Supplement: Supplementary file 2 — Table S1. [file BJH-209-324-s003.docx]

Supplementary table 1 – causes of hospitalization in patients

| **Patient** | **Underlying disease** | **Initial classifiation** | **Main reason for hospitalization** | **Outcome** |
| --- | --- | --- | --- | --- |
| 3 | sickle cell disease | D | orotracheal intubation due to acute respiratory insufficiency, liver enzymes > 1000 U/L | Discharge |
| 5 | sickle cell disease | B | abdominal pain and liver enzymes worsening on day 6 from DF onset | Discharge |
| 13 | aplastic anemia | B | cytopenia worsening on day 4 from DF onset | Discharge |
| 19 | sickle cell disease | C | thrombocytopenia worsening on day 4 from DF onset | Discharge |
| 20 | paroxysmal nocturnal hemoglobinuria | B | thrombocytopenia worsening with previous history of esophageal varices | Discharge |
| 23 | myeloproliferative syndrome | B | previous liver transplant, diarrhea and vomit worsening on day 7 from DF onset | Discharge |
| 29 | immunologic thrombocytopenic purpura | C | thrombocytopenia worsening with indication of intravenous immunoglobulin on day 1 from DF onset | Discharge |
| 32 | aplastic anemia | C | bleeding symptoms and thrombocytopenia worsening on day 2 from DF onset | Discharge |
| 34 | sickle cell disease | B | worsening of pain on day 3 from DF onset | Death |
| 35 | sickle cell disease | C | syncope on day 5 from DF onset | Discharge |
| 36 | sickle cell disease | C | acute renal injury | Discharge |
