# Supplementary material for: Outcomes of dengue infection in adults with underlying haematological diseases in Brazil during 2024 and 2025
Source: Br J Haematol. 2026 May 19;209(1):324–8. doi: 10.1111/bjh.70527 (PMC13340477; doi:10.1111/bjh.70527)
Supplement: Supplementary file 3 — Appendix S1. [file BJH-209-324-s001.docx]

16 - Kallás EG, Cintra MAT, Moreira JA, Patiño EG, Braga PE, Tenório JCV, Infante V, Palacios R, de Lacerda MVG, Batista Pereira D, da Fonseca AJ, Gurgel RQ, Coelho IC, Fontes CJF, Marques ETA, Romero GAS, Teixeira MM, Siqueira AM, Barral AMP, Boaventura VS, Ramos F, Elias Júnior E, Cassio de Moraes J, Covas DT, Kalil J, Precioso AR, Whitehead SS, Esteves-Jaramillo A, Shekar T, Lee JJ, Macey J, Kelner SG, Coller BG, Boulos FC, Nogueira ML. Live, Attenuated, Tetravalent Butantan-Dengue Vaccine in Children and Adults. N Engl J Med. 2024 Feb 1;390(5):397-408. doi: 10.1056/NEJMoa2301790. PMID: 38294972.

17 - Machado CM, Maiolino MG, Ramos JF, Carlesse F, Mendes AVA. Dengue Transmission by graft or blood transfusion in HCT recipients. J Bone Marrow Transplant Cell Ther. 2024 Apr 26; 5 (1), 230. doi: 10.46765/2675-374X.2024V5N1P230.
